# The draft genome sequence of *bla*_IMP-4_-carrying *Enterobacter soli* isolated from human blood

**DOI:** 10.1128/mra.00872-23

**Published:** 2023-12-08

**Authors:** Yu Feng, Zhiyong Zong

**Affiliations:** 1 Center for Pathogen Research, West China Hospital, Sichuan University, Chengdu, China; 2 Division of Infectious Diseases, State Key Laboratory of Biotherapy, Chengdu, China; 3 Center of Infectious Diseases, West China Hospital, Sichuan University, Chengdu, China; University of Maryland School of Medicine, Baltimore, Maryland, USA

**Keywords:** blood, IMP-4, *Enterobacter*, *Enterobacter soli*

## Abstract

*Enterobacter soli* is a Gram-negative rod characterized by its motile, non-spore-forming, and facultatively anaerobic nature. In this study, strain 140044, identified as *Enterobacter soli*, was isolated from human blood. The strain was sequenced, revealing a 5.37-Mb draft genome harboring the carbapenemase gene *bla*_IMP-4_.

## ANNOUNCEMENT

*Enterobacter soli* was named in 2011 (1), but its clinical relevance has not been reported. We provide the draft genome of *E. soli* strain 140044, isolated from the blood sample of a patient with acute pancreatitis and bloodstream infection at our hospital and considered as a pathogen. This study was approved by the hospital’s ethics committee, with the waiver of informed consent.

Bacteria grew from the blood sample in a BacT/Alert blood culture bottle (bioMérieux, Durham, NC) at 37°C for 24 hours aerobic incubation were inoculated on Luria agar at 37°C for 18 hours. The strain was recovered and plated on Luria agar as described above for purification. Single colonies were used for antimicrobial susceptibility tests (AST) and DNA preparation. AST were performed using the Vitek II automated system (bioMérieux), with results interpreted according to CLSI guidelines 2019 (2). After overnight culture in Luria-Bertani broth at 37°C with shaking, genomic DNA was prepared utilizing the QIAamp DNA Blood Mini Kit (Qiagen, Hilden, Germany). DNA was fragmented by sonication, and SPRIselect (Beckman Coulter, Brea, CA) was employed to target 350 bp inserts for library construction using the NEBNext Ultra II Kit (New England Biolabs, Ipswich, MA). The prepared libraries were loaded onto NovaSeq 6000 (Illumina, San Diego, CA) and sequenced in a 150-bp paired-end layout. Default parameters were used for all following bioinformatic pipelines unless otherwise specified. Generated read pairs (*n* = 6,681,542; coverage, 374×) underwent trimming of 10 bp from both ends using Cutadapt v4.4 (3), followed by removing adapters and low-quality (minimum quality, 15) bases using BBMap v39.01 (https://sourceforge.net/projects/bbmap). The quality-controlled reads were assembled using SPAdes v3.15.5 (4). Precise species was determined based on average nucleotide identity (ANI) with type strains of *Enterobacter* species using FastANI v1.33 (5). Antimicrobial resistance genes were identified using AMRFinderPlus v3.11.18 (6). Genome was annotated using PGAP v6.5 (7).

The draft genome of strain 140044 contains 5,366,569 bp (N50, 206,347 bp) in 98 contigs, with 53.15% G + C content. The strain has 97.33% ANI with *E. soli* ATCC BAA-2102^T^ (accession no. LXES00000000), establishing the species identification of *E. soli*. The draft genome contains 5,121 coding, 107 pseudo, 76 tRNA, 11 ncRNA, and 6 rRNA genes. Notably, carbapenem-resistant gene *bla*_IMP-4_ was identified along with several other genes predicted to mediate resistance to various antibiotics, including *aac(3)-IIg* and *aac(6′)-IIc* to aminoglycosides; *arr* to rifamycin; *bla*_ACT_, *bla*_DHA-1_, and *bla*_SHV-12_ to penicillins and cephalosporins; *qnrB4* and *qnrS1* to quinolones; and *sul1* to sulfonamides. In AST, strain 140044 exhibited resistance to carbapenems while was susceptible to amikacin, quinolones, and tigecycline.

**Fig 1:**
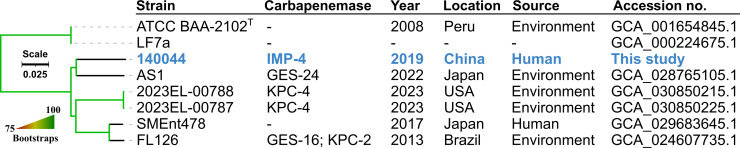
The phylogenomic tree of species *E. soli*. The phylogenomic tree was inferred based on concatenated SNPs that were identified by aligning individual assemblies against the chromosome of the *E. soli*-type strain, ATCC BAA-2102^T^. The tree was rooted at the midpoint. The reliability determined using bootstrap analysis was represented using a gradient color scale. Accompanying metadata were retrieved from the corresponding BioSamples and are displayed alongside the tree tips. Carbapenemases were predicted using AMRFinderPlus, and “-” indicates the absence of known carbapenemases.

A phylogenomic tree of strain 140044 and all available *E. soli* assemblies (*n* = 7) in NCBI were constructed using IQ-Tree v2.2.3 (8), from the single-nucleotide polymorphism (SNP) alignment generated by mapping genomes against the chromosome of LF7a (accession no. CP003026) using Snippy v4.6.0 (https://github.com/tseemann/snippy). Strains 140044 and AS1 share a recent common ancestor but with 32,147 SNPs (Fig. 1). Metadata and resistance profiles signal a global spread of *E. soli*, with concerns needing to escalate due to the emergence of metallo-β-lactamase in a clinical strain.

## Data Availability

The whole-genome sequence of 140044 was deposited in GenBank under the accession number JAVKYO000000000, along with its short reads deposited in the Sequence Read Archive available at SRR25949945.
